# Supplementary material for: Transfer RNA levels are tuned to support differentiation during Drosophila neurogenesis
Source: bioRxiv. 2024 Sep 6:2024.09.06.611608. Preprint. [Version 1] doi: 10.1101/2024.09.06.611608 (PMC11398488; doi:10.1101/2024.09.06.611608)
Supplement: Supplement 1 [file NIHPP2024.09.06.611608v1-supplement-1.pdf]

bioRxiv preprint doi: https://doi.org/10.1101/2024.09.06.611608; this version posted September 6, 2024. The copyright holder for this preprint (which was not certified by peer review) is the author/funder, who has granted bioRxiv a license to display the preprint in perpetuity. It is made available under aCC-BY 4.0 International license.

SUPPLEMENTAL TABLE 1

Isodecoder	Neuroblast	Neuron_me	log2FC	Origin	Isoaccepto	Diff_Exprs	q-value
mito:Cys-GCA	45045.38	20803.23	1.11	Mito	NA:Cys	NB Up	0.09
mito:Tyr-GTA	23475.05	7213.634	1.70	Mito	NA:Tyr	NB Up	0.09
Arg-TCT-2-1	12426.33	3840.326	1.69	Nuclear	Arg-TCT	NB Up	0.08
Ser-TGA-1-1	220526.7	123522.1	0.84	Nuclear	Ser-TGA	NB Up	0.06
Arg-TCT-3-1	6983.833	1779.569	1.97	Nuclear	Arg-TCT	NB Up	0.04
Arg-TCG-2-1	7972.896	4410.072	0.85	Nuclear	Arg-TCG	NB Up	0.79
Gln-TTG-2-1	11914.19	6549.584	0.86	Nuclear	Gln-TTG	NB Up	0.57
mito:Ser-GCT	77164.2	49711.86	0.63	Mito	NA:Ser	NB Up	0.48
Cys-GCA-2-1	2748.829	1573.917	0.80	Nuclear	Cys-GCA	NB Up	0.43
mito:Trp-TCA	17539.32	9328.135	0.91	Mito	NA:Trp	NB Up	0.37
SeC-TCA-1-1	63381.17	38962.99	0.70	Nuclear	SeC-TCA	NB Up	0.35
mito:Glu-TTC	6115.021	2878.862	1.09	Mito	NA:Glu	NB Up	0.32
mito:Phe-GAA	34053.7	18149.377	0.91	Mito	NA:Phe	NB Up	0.31
mito:Asn-GTT	52343.23	32638.3	0.68	Mito	NA:Asn	NB Up	0.30
mito:Gly-TCC	29539.39	13683.6	1.11	Mito	NA:Gly	NB Up	0.24
mito:Thr-TGT	4437.772	1585.99	1.48	Mito	NA:Thr	NB Up	0.23
mito:Leu-TAG	19413.05	7356.2342	1.40	Mito	NA:Leu	NB Up	0.22
mito:Arg-TCG	52598.46	24185.54	1.12	Mito	NA:Arg	NB Up	0.21
mito:Asp-GTC	9936.403	4340.493	1.19	Mito	NA:Asp	NB Up	0.14
mito:Met-CAT	47468.66	21332.39	1.15	Mito	NA:Met	NB Up	0.14
Pro-AGG-1-2	76862.797	136658.4	-0.83	Nuclear	Pro-AGG	Neuron Up	0.10
Pro-CGG-1-1	8382.3067	21618.82	-1.37	Nuclear	Pro-CGG	Neuron Up	0.08
Leu-CAA-1-1	30102.38	75745.6	-1.33	Nuclear	Leu-CAA	Neuron Up	0.08
Tyr-GTA-1-3	247820.8	536350.7	-1.11	Nuclear	Tyr-GTA	Neuron Up	0.08
Pro-TGG-1-2	139893.1	305538.3	-1.13	Nuclear	Pro-TGG	Neuron Up	0.03
Glu-CTC-1-1	111589.6	242531.1	-1.12	Nuclear	Glu-CTC	Neuron Up	0.02
Gly-GCC-1-2	678845.4	1383673	-1.03	Nuclear	Gly-GCC	Neuron Up	0.00
Thr-AGT-2-1	4542.351	7022.6391	-0.63	Nuclear	Thr-AGT	Neuron Up	0.64
Ser-AGA-3-1	1086.457	1837.095	-0.76	Nuclear	Ser-AGA	Neuron Up	0.55
iMet-CAT-2-1	16174.63	26826.99	-0.73	Nuclear	iMet-CAT	Neuron Up	0.47
Arg-TCG-3-3	3437.785	6200.77	-0.85	Nuclear	Arg-TCG	Neuron Up	0.42
Leu-TAA-4-1	29558.9	46968.14	-0.67	Nuclear	Leu-TAA	Neuron Up	0.32
Arg-TCT-1-1	92728.59	141178.8	-0.61	Nuclear	Arg-TCT	Neuron Up	0.32
Ser-CGA-1-4	30295.79	47351.05	-0.64	Nuclear	Ser-CGA	Neuron Up	0.30
Ala-TGC-2-1	30976.04	55567.96	-0.84	Nuclear	Ala-TGC	Neuron Up	0.28
Ala-TGC-1-1	25798.81	43295.1	-0.75	Nuclear	Ala-TGC	Neuron Up	0.28
Tyr-GTA-2-1	1587.959	3875.019	-1.29	Nuclear	Tyr-GTA	Neuron Up	0.27
Pro-CGG-2-1	41621.053	74905.3	-0.85	Nuclear	Pro-CGG	Neuron Up	0.26
Leu-CAG-1-4	128667.3	198541.2	-0.63	Nuclear	Leu-CAG	Neuron Up	0.16
Glu-CTC-3-7	120202	202069.7	-0.75	Nuclear	Glu-CTC	Neuron Up	0.14
Trp-CCA-2-4	39052.71	80169.57	-1.04	Nuclear	Trp-CCA	Neuron Up	0.13

bioRxiv preprint doi: https://doi.org/10.1101/2024.09.06.611608; this version posted September 6, 2024. The copyright holder for this preprint (which was not certified by peer review) is the author/funder, who has granted bioRxiv a license to display the preprint in perpetuity. It is made available under aCC-BY 4.0 International license.

Leu-TAG-1-2	133908.1	208317.7	-0.64	Nuclear	Leu-TAG	Neuron Up	0.11
Arg-ACG-1-8	782224.1	1122654	-0.52	Nuclear	Arg-ACG	No DE	0.06
Ile-TAT-1-1	129473.7	125896.2	0.04	Nuclear	Ile-TAT	No DE	1.00
Cys-GCA-3-1	9730.156	9192.401	0.08	Nuclear	Cys-GCA	No DE	1.00
Thr-TGT-2-5	152132.9	145860.8	0.06	Nuclear	Thr-TGT	No DE	1.00
Thr-CGT-1-2	247298.5	241977.6	0.03	Nuclear	Thr-CGT	No DE	1.00
Gln-CTG-1-1	63725.81	62206.32	0.03	Nuclear	Gln-CTG	No DE	1.00
iMet-CAT-1-5	138024.9	136322	0.02	Nuclear	iMet-CAT	No DE	1.00
Gly-GCC-2-1	135624.3	127744	0.09	Nuclear	Gly-GCC	No DE	0.99
mito:Leu-TAA	17087.498	17100.736	0.00	Mito	NA:Leu	No DE	0.99
mito:His-GTG	10028.69	8997.048	0.16	Mito	NA:His	No DE	0.99
Ser-AGA-1-1	2910.176	2458.195	0.24	Nuclear	Ser-AGA	No DE	0.99
Gln-CTG-4-1	47127.39	47941.04	-0.02	Nuclear	Gln-CTG	No DE	0.98
mito:Gln-TTG	7322.4517	6304.7450	0.22	Mito	NA:Gln	No DE	0.97
Ser-TGA-2-1	14398.79	12522.36	0.20	Nuclear	Ser-TGA	No DE	0.97
Met-CAT-1-2	367498.5	342648.1	0.10	Nuclear	Met-CAT	No DE	0.97
Asp-GTC-3-1	75636.08	78355.85	-0.05	Nuclear	Asp-GTC	No DE	0.97
Trp-CCA-1-2	7686.745	8295.933	-0.11	Nuclear	Trp-CCA	No DE	0.96
Asn-GTT-1-8	876720.7	833697.7	0.07	Nuclear	Asn-GTT	No DE	0.95
Leu-TAA-3-1	10221.79	8636.986	0.24	Nuclear	Leu-TAA	No DE	0.93
Asp-GTC-2-1	83171.1	90344.6	-0.12	Nuclear	Asp-GTC	No DE	0.91
Lys-TTT-1-1	102440.3	110878.8	-0.11	Nuclear	Lys-TTT	No DE	0.90
Arg-TCG-1-1	125770.4	136406.8	-0.12	Nuclear	Arg-TCG	No DE	0.90
Cys-GCA-1-1	181626.9	196316	-0.11	Nuclear	Cys-GCA	No DE	0.90
Cys-GCA-4-1	2190.373	1610.352	0.44	Nuclear	Cys-GCA	No DE	0.89
mito:Pro-TGG	29497.15	34342.913	-0.22	Mito	NA:Pro	No DE	0.89
Ala-CGC-1-2	74315.08	81566.01	-0.13	Nuclear	Ala-CGC	No DE	0.88
mito:Ile-GAT	96859.84	81312.46	0.25	Mito	NA:Ile	No DE	0.88
mito:Ser-TGA	9055.33	6581.245	0.46	Mito	NA:Ser	No DE	0.86
Thr-TGT-1-1	4662.956	3454.62	0.43	Nuclear	Thr-TGT	No DE	0.86
Ala-AGC-1-1	6225.478	7843.8214	-0.33	Nuclear	Ala-AGC	No DE	0.82
Lys-TTT-2-2	472738.1	496373.5	-0.07	Nuclear	Lys-TTT	No DE	0.82
mito:Ala-TGC	54073.17	43625.46	0.31	Mito	NA:Ala	No DE	0.82
Leu-TAA-1-1	9010.356	6755.4160	0.42	Nuclear	Leu-TAA	No DE	0.81
Pro-CGG-3-1	6034.229	7303.78	-0.28	Nuclear	Pro-CGG	No DE	0.80
Met-CAT-2-1	70818.8	85205.71	-0.27	Nuclear	Met-CAT	No DE	0.79
Val-AAC-1-1	15952.91	11833.05	0.43	Nuclear	Val-AAC	No DE	0.79
Gln-TTG-1-1	7308.036	8882.841	-0.28	Nuclear	Gln-TTG	No DE	0.78
Ser-GCT-2-2	15300.58	18548.73	-0.28	Nuclear	Ser-GCT	No DE	0.74
Gln-CTG-2-2	187372.7	214959.5	-0.20	Nuclear	Gln-CTG	No DE	0.74
Val-TAC-1-1	130657.1	152054.7	-0.22	Nuclear	Val-TAC	No DE	0.74
Thr-AGT-1-4	218453.1	243184.9	-0.15	Nuclear	Thr-AGT	No DE	0.71
Gln-CTG-3-1	102109.3	131847.6	-0.37	Nuclear	Gln-CTG	No DE	0.70
His-GTG-1-2	907612.9	1011253	-0.16	Nuclear	His-GTG	No DE	0.70

bioRxiv preprint doi: https://doi.org/10.1101/2024.09.06.611608; this version posted September 6, 2024. The copyright holder for this preprint (which was not certified by peer review) is the author/funder, who has granted bioRxiv a license to display the preprint in perpetuity. It is made available under aCC-BY 4.0 International license.

Leu-CAA-2-2	6292.322	9236.631	-0.55	Nuclear	Leu-CAA	No DE	0.69
mito:Lys-CTT	57946.53	41799.29	0.47	Mito	NA:Lys	No DE	0.67
Gly-TCC-2-1	97455.23	117302.8	-0.27	Nuclear	Gly-TCC	No DE	0.64
Leu-TAA-2-1	15025.51	19423.27	-0.37	Nuclear	Leu-TAA	No DE	0.64
mito:Val-TAC	66637.465	87398.72	-0.39	Mito	NA:Val	No DE	0.62
Phe-GAA-1-1	480520.7	400502.9	0.26	Nuclear	Phe-GAA	No DE	0.46
Val-AAC-2-1	215209	169734.7	0.34	Nuclear	Val-AAC	No DE	0.43
Arg-CCT-1-1	291887.9	340105.2	-0.22	Nuclear	Arg-CCT	No DE	0.42
Ser-GCT-1-1	7498.4255	10642.77	-0.51	Nuclear	Ser-GCT	No DE	0.41
Ser-AGA-2-2	676316.2	554538.4	0.29	Nuclear	Ser-AGA	No DE	0.39
Arg-TCG-4-1	67726.32	91453.61	-0.43	Nuclear	Arg-TCG	No DE	0.37
Val-CAC-1-1	44220.71	64324.12	-0.54	Nuclear	Val-CAC	No DE	0.37
Lys-CTT-1-10	674233	838799.2	-0.32	Nuclear	Lys-CTT-	No DE	0.32
Val-CAC-2-4	175025.6	239565.6	-0.45	Nuclear	Val-CAC	No DE	0.30
Glu-CTC-2-1	218255.4	300427.3	-0.46	Nuclear	Glu-CTC	No DE	0.27
Ala-AGC-2-3	180957.5	253549.8	-0.49	Nuclear	Ala-AGC	No DE	0.26
Ile-AAT-1-6	405138.7	315906.9	0.36	Nuclear	Ile-AAT	No DE	0.25
Glu-TTC-1-6	302209.4	398489.4	-0.40	Nuclear	Glu-TTC	No DE	0.16
Asp-GTC-1-9	990418.7	1344752.2	-0.44	Nuclear	Asp-GTC	No DE	0.16
Leu-AAG-1-4	234776.1	338430.7	-0.53	Nuclear	Leu-AAG	No DE	0.14
Gly-TCC-1-2	263229.8	387353	-0.56	Nuclear	Gly-TCC	No DE	0.11
